# Lived Experience Related to the COVID-19 Pandemic among Arabic-, Russian- and Somali-Speaking Migrants in Finland

**DOI:** 10.3390/ijerph18052601

**Published:** 2021-03-05

**Authors:** Eerika Finell, Marja Tiilikainen, Inga Jasinskaja-Lahti, Nasteho Hasan, Fairuz Muthana

**Affiliations:** 1 Faculty of Social Sciences, Tampere University, 33014 Tampere, Finland; 2 Migration Institute of Finland, 20500 Turku, Finland; Marja.Tiilikainen@migrationinstitute.fi; 3 University of Helsinki, 00014 Helsinki, Finland; Inga.Jasinskaja@helsinki.fi; 4 Tampere University, 33014 Tampere, Finland; Nasteho.hasan@hotmail.com (N.H.); Fairuzzz@hotmail.com (F.M.)

**Keywords:** COVID-19, pandemic, migrants, experiences, disease-related threats, health

## Abstract

Increasing research shows that migrants are disproportionately exposed to coronavirus disease 2019 (COVID-19). However, little is known about their lived experience and related meaning-making. This qualitative study maps COVID-19-related experiences among respondents from three migrant groups living in Finland: Somali-, Arabic- and Russian-speakers (*N* = 209). The data were collected by telephone interviews over four weeks in March and April 2020. Using inductive thematic analysis, we identified seven themes that illustrate respondents’ multifaceted lived experiences during the first phase of pandemic. The themes depict respondents’ difficulties and fears, but also their resilience and resources to cope, both individually and collectively. Experiences varied greatly between individuals and migrant groups. The main conclusion is that although the COVID-19 pandemic may be an especially stressful experience for migrant populations, it may also provide opportunities to deepen cooperation and trust within migrant communities, and between migrants and their country of settlement. Our analysis suggests that cooperation between local authorities and migrants, trust-building and effective information-sharing can foster positive and functional adaptations to disease-related threats and changing social environments.

## 1. Introduction

### 1.1. Severe Acute Respiratory Syndrome Coronavirus 2 (SARS-CoV-2) as a Special Risk for Migrant Populations

Coronavirus disease 2019 (COVID-19), caused by the severe acute respiratory syndrome coronavirus 2 (SARS-CoV-2), became a global health threat at the beginning of 2020. The pandemic revealed vulnerabilities and inequalities with regard to racialised and ethnic minority communities. Several reports indicate that COVID-19 infections and related mortality rates are higher among such communities than in the majority population [1,2]. Social and economic conditions such as overcrowded housing, poor access to health services, reliance on public transport, concentration in certain jobs, weak social security (e.g., sick leave allowance), and poor access to other resources have been seen as key factors to explain these disproportionally high rates. In addition, it has been argued that pre-existing health disparities, such as chronic medical conditions, contribute to severe forms of the disease in this population [2].

Alongside physical health, migrant (we use the term ‘migrant’ to refer to both migrants and refugees) populations’ psychosocial well-being can be seriously affected by COVID-19. Sieffien and colleagues [3] suggest that migrants’ higher vulnerability to psychological distress during the pandemic is due to their higher exposure to pre-migration traumatic events, lack of immediate family or other supportive social networks, and poor access to healthcare. Migrants often experience prolonged separation from their families, which creates transnational worry and negatively affects their well-being [4]. In addition, lockdowns due to the pandemic [5] and information deprivation due to language barriers [3] may further exacerbate migrants’ mental health problems and lead to difficulties receiving adequate help. Despite these concerns, empirical research on the psychological and social impacts of the COVID-19 pandemic on migrants’ well-being is still relatively scarce. One of the few exceptions is the study by Barbato and Thomas [6] among Italian workers living in the United Arab Emirates (UAE), which found that COVID-19 put them at heightened risk of post-traumatic stress disorder symptoms, depression and anxiety (see also [7,8]).

Finally, past or current experiences of discrimination may also amplify migrants’ vulnerability during the pandemic [3]. After the outbreak of COVID-19, there were signs of an increase in hate crimes, racism and prejudice against migrants, particularly those whose countries of origin were epicentres of the disease [9]. Thus, Tabri and colleagues [10] found that perceived existential threats stemming from COVID-19 manifested in prejudice against Chinese people in the United States. Litam and Oh [11] found that COVID-19-related racial discrimination decreased Chinese Americans’ life satisfaction via increased levels of depression (see also Wang et al. [12] from France). The role of official and social media in increased prejudice has also been documented (e.g., Croucher et al., [13]).

It is important to note, however, that these populations are heterogeneous in terms of socio-cultural backgrounds and resources, and migration histories. They may also utilise culturally specific coping resources. For example, the Islamic faith has been found to provide Muslims with hope and comfort in the midst of uncertainties and fears caused by COVID-19 [14]. Hence, there may be significant differences in how adversities are experienced and faced by different migrant groups and individuals, and how much distress they cause.

### 1.2. The Present Study

The aim of this study is to describe the lived experiences and meaning-making of Russian-, Somali- and Arabic-speaking migrants in relation to COVID-19, and their everyday responses to the threat of the virus. Although migrant populations’ vulnerability during the pandemic has been acknowledged in the literature, most of the previous research that we have traced has been quantitative (for an exception, see e.g., [8,15]). Our study fills this gap. To understand how the COVID-19 pandemic affects migrants and what kinds of help are needed, first-hand experiential accounts are essential. Moreover, in order to understand the cultural specificities of responses and develop well-targeted interventions, we need a profound understanding of collectively shared experiences as well as experiential differences among individuals and groups.

Our study is located in Finland, where COVID-19 arrived relatively late. On 16 March 2020, the Finnish government declared a state of emergency over the SARS-CoV-2 outbreak. Despite the low infection and mortality rates, relatively strict restrictions were instituted: teaching was moved to remote learning, nightclubs were closed, and all gatherings of more than nine persons were forbidden. However, people were allowed to spend time outdoors and use public transport. The first wave remained fairly well controlled in Finland; the number of COVID-19-related deaths (328 people until 1 July 2020) was relatively low [16].

In this study, we focus on the lived experiences of three migrant groups during the first wave: Russian, Somali and Arabic speakers. These groups represent the biggest migrant communities in Finland, which is characterised by a small but growing migrant population (8%) [17].

Until the collapse of the Soviet Union in the 1990s, the Russian-speaking population in Finland was quite small. Today, Russian speakers represent the biggest migrant group: 22% of all foreign-language speakers speak Russian as their mother tongue [17]. At the end of 2019 there were almost 81,600 Russian speakers in Finland [18]. Most Russian speakers came to Finland either as return migrants of Ingrian-Finnish background (Ingrian Finns are descendants of Finns who settled in the St. Petersburg area in Russia called Ingria in the 17th century, when Finland and Ingria were both parts of the Swedish Empire), for work, or due to marriage to a Finnish citizen. Russian-speaking migrants are highly educated, but their employment rate is considerably lower (61%) than that of native Finns [19]. There are many reasons for the Russian-speaking community’s socio-economic disadvantage, but prejudice and discrimination have been seen as critical (see e.g., Larja et al. [20]).

The Somali-speaking population started to migrate to Finland about 30 years ago, when civil war broke out in Somalia at the end of the 1980s. Until recently, Somali speakers constituted Finland’s largest group with a refugee background. At the end of 2019 there were almost 22,000 Somali speakers in Finland. They mostly comprise people who arrived as asylum seekers or through family reunification procedures. This population group is generally young: approximately 44% are under 20 years of age, and less than 9% are over 49 [18]. Several studies have reported that Somalis in Finland commonly face discrimination and racism in their everyday lives [21] and in the labour market [22]; their unemployment rate has remained high.

The number of Arabic speakers in Finland has risen more than threefold in the past 10 years. At the end of 2019, their number was almost 32,000, whereas in 2009 it was under 10,000. The main reason for the increase is the peak in asylum seekers arriving from Iraq and Syria in 2015. Arabic speakers have also arrived due to marital reasons and as students from North Africa, Sudan and the Middle East. Currently, about 61% of Finland’s Arabic speakers are male. Over 36% are under 20 years of age, and 12% are 50 or over [18]. Like Russians and Somalis, Iraqis have also faced challenges in getting employment [22].

Finland provides a unique context to study the lived experiences of these migrant groups. It is a Nordic welfare state where high-quality public health services are available for free or at low cost for all residents. Finnish people in general have a high level of trust in public administration [23], and societal trust also seems to be high among migrants living in Finland. According to one recent population study, almost 66% of people of foreign backgrounds trusted the public health services, compared with almost 53% among the whole population [24]. To date, most COVID-19-related research among migrant populations has been conducted in countries with lower societal trust. This is therefore another way in which our study contributes to the research.

## 2. Materials and Methods

### 2.1. Recruitment and Procedure

The data collection had two objectives: to produce scientific knowledge, and to inform the authorities about whether and how migrants receive information about the COVID-19 situation in Finland. The first author informed the relevant authorities of findings in three bulletins during the data collection.

The first interviews were conducted a week after the Finnish government had declared a state of emergency. The recruitment had started a few days earlier, using snowball sampling. When the crisis hit Finland, there was a great and urgent need for information. Therefore, we did not have time to apply for a representative sample from the Digital and Population Data Services Agency of Finland, where it could take a long time to obtain a sample. In addition, using snowball sampling, we had the opportunity to reach even those respondents who generally do not want to be contacted and who do not allow their telephone numbers to be given for marketing or research. This method has been widely used to study marginalized or hard-to-reach populations in other contexts too [25]. The interviewers first interviewed people they knew from the target population. Then they asked if these respondents knew other people who belonged to this population and would like to join the research. Usually the respondent first contacted these people and asked if their telephone numbers could be given to the interviewer. After that the interviewer contacted them. In addition, respondents were recruited through social media (e.g., Facebook) and people who had a central position in the language group.

Our target population was migrants who spoke Arabic, Russian or Somali as their native language and were aged 50 or over. The focus was on the older population because age predisposes individuals to serious forms of COVID-19 disease and, therefore, this age group’s experiences and information acquisition were a high priority. However, interviewers were also permitted to interview younger people if they had problems reaching the older population (*N* = 61, 29% of the sample). 

Telephone interviews were conducted between 23 March and 17 April 2020 by three female research assistants who were native speakers of Somali, Arabic and Russian and also had excellent Finnish-language skills. At the beginning of the interview, all respondents provided their informed consent. The questions addressed worries associated with the coronavirus pandemic, information acquisition related to the pandemic (in order to keep the data set to a manageable size, we do not provide information here about the different newspapers (e.g., *Helsingin Sanomat*), TV channels and news agencies (e.g., the British Broadcasting Corporation (BBC)), social media platforms (e.g., Facebook), workplaces or study environments from which respondents received their information. This large data set will be reported elsewhere) how people protected themselves from the virus, whether they trusted the Finnish government, to whom they could turn for help, what kind of help they needed, and any other thoughts about the issue. At the end of the interview, background information was requested. Respondents’ answers were written down and translated into Finnish. Interviews were not recorded because we wanted to avoid placing extra stress on our respondents, many of whom were not used to participating in research. The interviews lasted between 10 and 30 min. Some answers were short (‘*yes, this is a worldwide problem*’), while other respondents gave longer and more detailed answers (approximately 100 words).

### 2.2. Respondents

In total, 209 respondents were interviewed in Arabic (*N* = 64), Russian (*N* = 68) and Somali (*N* = 76) languages, except one respondent who wanted to be interviewed in Finnish. To secure his/her anonymity, in Table 1, based on the cultural background, this respondent is categorised with those who were interviewed in Arabic. Most of those interviewed in Arabic had been born in Iraq (89%). The rest had either Syrian (*N* = 5) or Palestinian (*N* = 1) backgrounds. Given that the responses of these six respondents where similar to those who were born in Iraq, we did not exclude them from the sample. In addition, migrants’ language skills rather than their ethnic or national background have guided the efforts of Finnish authorities in spreading COVID-related information in migrant communities. One Arabic-speaking respondent did not report his/her country of origin. Almost all respondents interviewed in Somali reported that they had been born in Somalia or Somaliland (95%). One participant had been born in Ethiopia. The remaining 4% did not reveal their native country. All the respondents interviewed in Russian had been born in the former Soviet Union.

Our sample’s demographic characteristics are reported in Table 1. Most respondents were female (*N* = 149, 71%) and older than 49 (*N* = 146, 70%). Two respondents did not provide their age. About one third of respondents (*N* = 63, 30%) reported that they understood the Finnish language well. Only 35 respondents (17%) reported that they lived alone.

### 2.3. Analytical Strategy

We analysed the responses following the five phases of thematic analysis described by Braun and Clarke [26]. This was an active, organic process. First, the data were read carefully, and preliminary descriptive codes were created by three authors and one research assistant working in close cooperation. In the second and third phases, the first author systematically and inductively recoded the data relevant to the research topic. The same text could be coded with several codes. An analytical unit varied between a few words and a few sentences. Then raw codes that dealt with the same issues were categorised under the same key code, and the first author reanalysed the data several times, using these key codes and inserting new ones if needed, in an iterative process. Preliminary subthemes and key themes were identified, and then evaluated and discussed by the rest of the team. In the fourth phase, the first author reanalysed the data to ensure that each theme made a coherent and distinctive whole, and that the themes covered the data exhaustively. The reliability of the analysis was ensured by the fourth and fifth authors, who systematically checked the data at the level of key codes. There was disagreement in 8 cases. In the last stage, the final themes were named, and their contents were clarified. The first author translated the extracts from Finnish into English. Alongside each extract, we report the respondent’s age and migrant group. In order to secure their anonymity, we do not report respondents’ gender unless necessary.

## 3. Results

### 3.1. The Spectrum of Different Experiences

We identified seven key themes. The first three depict the difficulties, negative experiences and fears our respondents faced during the beginning of the COVID-19 crisis in Finland. The next three discuss the different ways they coped with the situation. The final theme describes how they gave the crisis a new, more comforting meaning. Each key theme has at least two subthemes. The key themes and subthemes are presented in Table 2. The frequencies of each theme are given for each migrant group. The list of key codes is reported in Appendix A.

Although some key themes and subthemes seem to contradict each other, many of them might be identified within a single interview. In order to illustrate this, we provide an example below. At the beginning of one interview, the respondent (Arabic, aged 40–49) said that he was not worried about the coronavirus pandemic:

*I have no worries whatsoever. I live normally—as I have always lived. I still go out and meet people, and I don’t care about it or fear the virus in question. (Coded as: There is nothing to be afraid of, I live a normal life; key theme 4, subtheme 4)*

But later in the interview, he continued:

*The other day my ex-wife contacted me and reported that my child has a fever and symptoms of flu, so it caused me a lot of stress. (Coded as: Worry about friends and family; key theme 3, subtheme 1.) I was reading the Koran and remembering God in that moment. (Coded as: Trusting in God and being a good Muslim; key theme 4, subtheme 1)*

Thus, our respondents experienced the pandemic in multiple ways: they could report being fearful and unworried, trustful and distrustful, lonely and connected at the same time. Below we describe the themes in detail.

### 3.2. COVID-19 Infection as a Health Threat

The first theme discusses the fear and anxiety associated with COVID-19 disease and coronavirus infection. Most respondents perceived coronavirus as an unknown and dangerous health threat. Two respondents below depict their feelings:

***Extract 1.*** *Yes, I am very worried, because we do not know where it has come from, and it is only a matter of time before we get infected. When I heard that it was in China, I got scared, and it had not yet arrived in Finland at the time. [...] It’s all because of fear. I’m very scared. That virus has no medication or vaccine, which scares me more. (Arabic, 50–59)*

***Extract 2.*** *I am worried about the symptoms, as they greatly resemble the symptoms of normal flu. I was just short of calling an ambulance yesterday, because my mouth was very dry, and I was feeling bad, and I did not know if it was one of these symptoms. (Arabic, 69+)*

These two extracts illustrate many of the factors that instilled fear and worry in respondents. They were afraid of being infected, and worried about the fact that the symptoms resembled those of flu. They also mentioned the lack of proper treatments or vaccines, and concern about the origin of the virus. For some the fear was intense, as seen in the extracts above. In extract 1, a middle-aged respondent explains that she is very concerned about potential infection. In extract 2, an elderly respondent describes her experiences relating to the odd symptoms. Later in the interview she continues: ‘*Everything is available, but my mental health is in a very poor state. The last time I experienced similar anxiety was in 1991 in Iraq during the war*’. This interviewee parallels the anxiety caused by the fear of disease with her previous war experiences. Previous traumatic experiences may have made some respondents more vulnerable to the stress caused by the pandemic situation and related risks, although only a few respondents referred to their previous war experiences. Worry about one’s own health was especially common among Arabic speakers.

### 3.3. Being Vulnerable in Finland

In addition to being a health threat, COVID-19 disease produced an exceptional and uncertain situation that affected the whole society, producing fear, stress and worry in equal measure: ‘*The world will change completely, as well as our future. This lack of awareness is really distressing*’ (Russian, 30–39). The second theme discusses the experience of being vulnerable in the face of the COVID-19 pandemic, as a migrant in a country whose institutions one does not properly know or fully trust and whose language can pose difficulties.

Respondents reported being worried about the pandemic’s impact on society and the economy, and some were distressed due to social isolation. Worries about economic and social problems were especially common among Russian speakers; for some, the emotional burden was so great that they even said they needed psychological support. Mainly Somali speakers reported that social isolation was stressful to them.

In the interviews, worries concerning health and material well-being could occur at the same time as a distrust of Finnish authorities and an experienced lack of support or information. In addition, some respondents lacked supportive social networks. Below, two Russian speakers and one Somali speaker depict their experiences of vulnerability.

***Extract 3.*** *I lost my job, the rent worries me a lot, how I will get it paid and other bills. Material help is the most important at this stage. This scares me more than the virus itself. Kela (The Finnish Social Insurance Institution) does not react to this situation of mine. I have no faith in the future. All treatments cost a lot, how do I pay for them? Hope has disappeared completely. (Russian, 40–49)*

***Extract 4.*** *I am basically by myself. I think that when one calls the doctor now, they will take more seriously a person, who speaks the Finnish language well, than me. That is, a person that speaks the Finnish language worse. (Russian, 40–49)*

***Extract 5.** I can’t say where I can get help. If I got a service in my own language, then I might know who to contact. […] News [readers] speak quickly. Difficult to understand them. Finnish is the most difficult language […]. (Somali, 60–69)*

In extract 3, the respondent says that she has just lost her job and is afraid of receiving no monetary help from the state. In addition, she is afraid that she will be unable to pay for medical care. This respondent is alone in Finland, without supportive relatives: ‘*There is no one. I am alone with my child*’. The fear of poverty and the experience of being without support manifested as deep despair (‘*hope has disappeared completely*’). In extract 4, the respondent explains her fear of discrimination due to her poor language skills and how she feels that she can rely only on herself. In extract 5, the respondent reports that she does not know where to seek help due to her restricted Finnish-language skills.

Such experiences of being left without support or information were shared by many other respondents. They said that there was no one that could help them, and they did not know how they could receive help or to whom they should turn. In many cases, poor Finnish-language skills were associated with these experiences: in particular, some Somali and Arabic speakers said that they could not receive information directly from the authorities because they did not understand Finnish news reports or announcements. This could also lead to feelings of exclusion. The lack of information in Arabic and Somali languages about COVID-19 in Finland was especially apparent at the beginning of the crisis (note that Finnish Broadcasting Company produced Russian language news). However, the situation changed fast, and when our data collection ended, information in Arabic, Russian and Somali languages (among many others) could be found relatively easily.

Extracts 3 and 4 also illustrate some respondents’ difficulty in trusting the Finnish authorities’ ability or motivation to help or control the crisis. This distrust of Finnish authorities was related to the perceived lack of resources and decision-making power, insufficiently strict measures, poor-quality healthcare, or the uncontrollable nature of epidemic. Although this distrust was shared by all migrant groups, it was especially common among Russian speakers.

Finally, these extracts illustrate how the combination of distrust in society and a perceived lack of (material and immaterial) resources can make migrants especially prone to stress, and might prevent them from seeking help and support during times of crisis. Therefore, they may experience the pandemic as an especially critical life event: ‘*I am not afraid of death because of my history, but because I am in a foreign country. This causes fear*’ (Arabic, 69+).

### 3.4. Communal and Multilocal Worries

While the previous theme discussed the fear, worry, distrust and lack of support our respondents experienced individually in Finland, this theme captures their fears for the well-being of their transnational families (in both Finland and other countries) and ethnic communities, as well as their concerns related to the situation in their countries of origin. In other words, this theme sheds light on the multilocal and collective nature of migrants’ worries during the COVID-19 pandemic and the extra burden this places on them.

Multilocal worry is concretely illustrated in the extract below:

***Extract 6.*** *I am worried about the situation in Finland because everything has stopped. Schools, jobs. Children are at home. This is a difficult time for us because we are a big family. I am worried about my home country because my family, parents and friends live there. There’s no good healthcare there. I have friends who passed away because of coronavirus. They had shortness of breath and were not put on a ventilator. (Somali, 60–69)*

This respondent reports that he is worried about the situation in both Finland and Somalia. He explains that in Finland work has stopped and the schools are closed, and this time is difficult for his family; implicitly he also refers to challenges faced by the family in overcrowded housing. Simultaneously, he is worried about friends and relatives in Somalia because of the country’s low-quality healthcare and its lack of other resources. In addition, due to unemployment, financial remittances sent to family and kin in Somalia may be at risk. Finally, multilocal worry is manifest in the fact that he has already lost friends due to COVID-19 in Somalia. This double worry was similarly expressed by another Somali speaker: ‘*I’m worried about my homeland. I am worried about both [countries]. We live here, and my family lives in [my] homeland*’ (50–59).

Distress about the well-being of family and friends was common among respondents. Travel restrictions and visa problems were likely to intensify this stress, as illustrated below:

***Extract 7.*** *I have elderly parents, and I am very worried about their health. Their visas will run out soon, and I’m frightened about what happens then. I wouldn’t want them to go back to Russia, because at the moment it’s the same thing as sending them to their deaths. (Russian, 50–59)*

The respondent explains that her parents’ visas are expiring. Due to the travel restrictions, she is not sure whether her parents can stay in Finland. She finds the situation stressful because the COVID-19 infection rate is higher in Russia than in Finland, and she feels that if her parents have to return to Russia, they will likely become infected and die.

However, worry about other people was not restricted to one’s family and friends in Finland and elsewhere. Among Somali speakers it also extended to the whole ethnic community. As one respondent told us: ‘*I heard about Sweden. There, 12 people with Somali backgrounds passed away*’ (Somali, 69+). Three weeks later, Helsinki’s municipal authorities publicly announced that the Somali population was overrepresented in Finland’s infection cases too (see Helsinki City [27]). This situation increased the worry among many of our respondents: ‘*I’m not worried about myself, I’m worried about the whole people. Especially about my community, who have had challenges following the authorities’ instructions*’ (Somali, 60–69). However, not everyone expressed their concerns so directly. Some expressed their worries by stating their wish for more information about COVID-19 to be shared with their community: ‘*There are many people with Somali backgrounds who do not have children and cannot speak the Finnish language and can’t read. They hope very much to get information from the TV*’ (Somali, 50–59). It is notable that Somali speakers were not the only ones who expressed the wish for more information to be distributed to migrants; some Russian speakers also did so.

In addition to the concerns described above, many respondents reported being concerned about their country of origin in general, which added to their stress.

***Extract 8.*** *I never had hypertension problems before, but now I’m suffering from that constant stress. I am more concerned about the situation in Iraq, and it is part of why I suffer from hypertension problems, because the situation in Iraq is very bad. Finland is a small country, so the virus can be controlled, whereas in Iraq the situation can escalate very easily. (Arabic, 40–49)*

In this extract, the respondent explains that stress about the situation in Iraq is causing her hypertension. This extract also demonstrates that multilocality leads easily to comparisons between one’s countries of descent and settlement. Thus, although respondents might report little anxiety about the situation in Finland, Arabic- and Somali-speaking respondents in particular often reported that they were concerned about the situation in their countries of descent and relatives and friends living there.

### 3.5. Living with the Threat of the Virus in the Everyday

Regardless of the concerns the COVID-19 pandemic caused, many respondents emphasised the importance of avoiding panic or overwhelming fear. As one respondent explained in Russian (50–59), ‘*I think at the moment that great panic is causing more harm than the virus itself*’. Thus, many respondents perceived stress about coronavirus as unhealthy in itself, because stress reduced immunity or caused other problems. Protecting oneself from fear was a way to protect oneself from the virus. Thus, while the first three themes focused on the psychological and practical challenges our respondents faced during the pandemic, this fourth theme depicts how respondents tried to face and control the fear these challenges evoked in them, by either downplaying the risk, avoiding thinking about it, caring for their health, doing their best, turning to God and/or accepting their destiny: ‘*Everyone dies when their time comes. Great fear produces problems*’ (Somali, 50–59).

One common way to cognitively cope with the threat in everyday life was to emphasise that there was no need to be worried because COVID-19 was like ordinary flu (‘*there are no worries whatsoever, as I consider it is normal flu*’, Arabic, 50–59). Another way to escape the fear was to avoid news about COVID-19 or avoid thinking about the threat (‘*I avoid thinking, because I know I belong to the risk group, so I try to avoid it*’, Arabic, 69+). The belief that the pandemic would end in due course also helped to preserve peace of mind. In addition, Arabic-speaking respondents in particular blamed the media or other people for feeding the fear. They explained that the media was exaggerating the threat, which increased fear and stress (‘*television causes stress, because the issue is dramatized there a lot*’, Arabic, 40–49). Two respondents also engaged in conspiracy thinking by explaining that coronavirus was a human-made virus and therefore there was no need to be afraid of it.

Another important way to manage the situation was to care for one’s own health. Almost all respondents said that they either paid attention to hygiene or avoided contact or both. Only a few reported that they relied on nutrition alone or did not protect themselves at all. Some respondents highlighted that caring for good hygiene was a key part of being a good Muslim. Hence, for some, health-related instructions gained deeper meaning through religious interpretations.

***Extract 9.*** *We are Muslims, and our religion teaches us how one needs to act in situations like this. When the Prophet Mohammed was alive, an illness broke out, and then places were shut, and no one was allowed to go away or leave. God asks that we trust him, but we must act in the best way so that one can prevent [illness] in every way. (Somali, 50–59)*

For most Somali and many Arabic speakers, turning to God and Muslim identity was an important way to make sense of and cope with the situation. They said that they prayed to God, asked for his protection against COVID-19, and accepted his will without fear; because their destiny was already written, it could not be changed and, therefore, there was no reason to be afraid: ‘*I don’t have any fear about this. Everything is written. What will come, will come*’, explained one Somali speaker (69+).

Nevertheless, to rely on God’s will did not dispel all worries. As one respondent said: ‘*Thank God, everything that God brings, as a Muslim I consent. But as a human being, I’m worried*’ (Somali, 60–69). These words demonstrate the dualism between being worried as a human being and not being worried as a devout Muslim. A similar dualism can be detected in relation to faith and duty, reflecting the Islamic understanding of disease and suffering in general: even if God has created illness, he has also created medicine, and a human being should do his or her best to be patient and seek a remedy [28]. In the same vein, when respondents expressed a belief in God’s will, they might also underline people’s responsibility to protect themselves and others from the virus. Thus, for them faith did not reduce individual agency or responsibility: ‘*I do my best, but God protects*’ (Somali, 50–59).

Among Somali-speaking respondents, and to some extent also among Arabic speakers, religion thus appeared as a protective factor: it helped them to give meaning to the disease and suffering the virus caused, but it could also support protective measures and resilience against the virus (cf. Bentley et al. [14]).

### 3.6. Building Trust and Cooperation with Finnish Society

The COVID-19 crisis made visible the state’s role as the guardian of citizens’ and residents’ welfare. Thus, when asked what kind of help they might need and to whom they would turn to receive it, a large majority referred to the help of the authorities—although some expressed this more as a wish than a fact: ‘*I pray that it [infection] does not come, but if it does come I hope I will get the support that other people are entitled to*’ (Somali, 60–69). They mainly referred to medical help, but they also pointed out that they needed economic support, either immediately or potentially in the future; they asked for psychological support and hand sanitiser; they hoped that someone would buy and deliver food for them if they could not go grocery shopping; they asked for more information for themselves. Thus, the fifth theme discusses how respondents dealt with the crisis by relying on and trusting the authorities, following their instructions and building cooperation: ‘*The Finnish state takes care of us. If anything happens, I’ll go to the hospital*’ (Arabic, 60–69).

Although there were also critical voices (see theme 1), most respondents trusted Finnish society’s ability to handle the COVID-19 crisis, at least to some extent. They provided several reasons for this. Mainly Arabic and Somali speakers explained that the quality of the Finnish healthcare system was high, Finland was a small country with good know-how, Finns followed instructions, and Finland was doing better than other countries with COVID-19 (‘*in Finland, there are only 28 dead. In other countries, more than hundreds*’, Somali, 60–69). In addition, many respondents from all migrant groups were satisfied with the measures established by the Finnish government and believed that the authorities (four Arabic-speakers also reported that they received help from a Finnish non-governmental organisation (NGO)) cared about people, helped them, and tried their best:

***Extract 10.*** *I see that the Finnish authorities are doing their best. The Finnish state is a good state because they think about the best for residents. They do everything possible. Finland’s healthcare is one of the best. (Somali, 50–59)*

These positive experiences made it possible for respondents to build trust in Finnish society: ‘I did not believe before, or there was just a small belief that the government is able to handle this. But now I believe that they are trying hard’, explained one Russian speaker (50–59). He also referred to the coronavirus announcement sent to citizens via SMS in the spring: ‘Text messages from the state bring a sense of security’. Thus, trust in society and the authorities helped respondents to live with fear and worry: ‘I am not afraid because I trust the Finnish authorities’ (Arabic, 69+).

Another way to deal with the situation was to follow authorities’ instructions. As explained in theme 4, most respondents said that they either paid attention to hygiene or avoided contact or both. However, such behaviour was not only to protect oneself or one’s family—for some, it was also a cooperative project between residents and the authorities (‘*when I stay at home, I help the government with this situation*’, Russian, 40–49), and it reflected the legitimacy of and trust in the Finnish state and government (‘*I follow the orders, so I feel that I am safe*’, Arabic, 69+). Below, a Somali speaker explains why the Finnish authorities can handle the pandemic satisfactorily:

***Extract 11.*** *I believe that they can. I see that Finnish authorities are doing their best. So, if we cooperate with the authorities, it is possible. (Somali, 60–69)*

This extract illustrates how the pandemic seemed to emphasise the understanding that crisis management requires cooperation between different actors in society. The pandemic was not only a source of threat and worry, but also an opportunity to see oneself as an active agent in Finnish society—someone who has obligations towards the state and who has the right to get help when needed:

***Extract 12.*** *The authorities help people very much, and we need their support if we get sick. Me too, I am also part of this society. (Somali, 50–59)*

### 3.7. Supporting Each Other and Taking Collective Action

Although the authorities are an essential source of help, the most immediate help is usually received from families and communities. The sixth theme discusses the social support received from families and ethnic communities, and the collective action that emerged to protect them. The respondent below explains how she and other mothers created shared norms to protect their ethnic community.

***Extract 13.*** *Children don’t visit each other. This is what we mums agreed with each other, so that none of our children get the chance to go out because their friends are out. There are few of us here. (Somali, 50–59)*

In all migrant groups, family members and friends were the most important source of social support. They played an essential role in translating and sharing information, supplying food, and helping with bureaucracy. In addition, friends and family living in other countries provided information and psychological support: ‘*I receive information from my relatives, who live all around the world*’ (Arabic, under 40).

In addition to close networks of family and friends, many Somali- and Arabic-speaking respondents spoke about the active members of their ethnic/language communities who helped them, especially by sharing information: ‘*We have friends on Facebook who also share information about this. They help us understand the authorities’ instructions by translating Finnish texts into Arabic*’ (Arabic, 50–59). Thus, COVID-19 created collective action and mobilised people within communities. The ethos of working together against the coronavirus threat was most visible in the responses of Somali speakers, who referred to the activity of many community members: ‘*In our community many have taken on a role and worked hard, like our community’s doctors and imam*’ (Somali, 60–69). Some also highlighted that they had participated themselves by translating and sharing information: ‘*We have, as a Somali community, determined together to assemble the folks and communicate to our community the best and most reliable information*’ (Somali, 60–69).

This collective action was perhaps related to the fact that the Somali population had significantly higher rates of COVID-19 infection in Finland than the rest of the population in March and April 2020 [27], and the community took an active role in cooperating with the authorities to improve the situation. Below, a respondent explains how she received information about the COVID-19 situation in Finland.

***Extract 14.*** *Bulletins are sent from mosques, a Somali doctor/nurse in Finland talks online about coronavirus. S/he encourages us to listen to the authorities’ instructions. (Somali, 60–69)*

This hard work yielded good results: ‘*Now our community takes this seriously and pays more attention to the authorities’ instructions*’. By the end of our data collection, all our Somali-speaking respondents knew where to seek help if needed and how to protect themselves properly. In June 2020, daily COVID-19 cases fell close to zero (<20) in Finland [16]. Below, two respondents explain their feelings about the process, which varied from sorrow that the Somali community had received negative publicity due to COVID-19 infection rates, to pride and empowerment that members of the group had raised their voice and protected their community:

***Extract 15.*** *Sometimes I feel like the media puts Somalis in a bad light, and this makes me sad. (Somali, 60–69)*

***Extract 16.*** *It’s great to see how our young people are trying to work to make our voice heard. We are part of this society, and I am grateful that our young people are getting into this job. (Somali, 50–59)*

### 3.8. Globally Shared Worry and Cross-Border Solidarity

Our final theme discusses how global worry turned into a shared experience that gave the crisis a new, more comforting meaning. Respondents’ worries were not restricted to their immediate environment, family, community or country of origin; many of them also perceived that COVID-19 was a global crisis: ‘*I am certainly worried, because it is a pandemic that has spread everywhere*’ (Arabic, 60–69); ‘*I am not worried about [my] homeland. I am worried about the whole world*’ (Somali, 50–59). However, a global crisis also has the potential to become a globally shared experience (‘*I don’t think anyone would not be worried*’, Somali, 50–59) that can unite people instead of dividing them and make mutual interdependency salient (‘*Finland is part of this world. It’s no different from other countries. Everyone is in the same boat*’, Somali, 50–59). Below, two respondents explain their feelings:

***Extract 17.*** *I feel that it has taught us all that the virus in question doesn’t look at age, race or any other factor affecting the individual’s life—everyone has the same risk of getting it, so we should all support each other. (Arabic, 40–49)*

***Extract 18.*** *Actually, I’ve noticed that coronavirus has increased human equality. It doesn’t look at skin colour, black or white, Muslim, non-Muslim, going to work or not. I hope this equality will continue. [...] But from this pandemic we can learn human equality. (Somali, 30–39)*

In these two extracts, the respondents explicitly state that the virus treats all people equally. It does not consider age, religious belief, ethnicity or economic background and, therefore, everyone faces the same risk of infection. From this observation, both respondents argue, we have an opportunity to learn something important. In extract 17, the respondent states that people should support each other; in extract 18, the respondent hopes we will learn from the pandemic that people are equal. Thus, they give COVID-19 a meaning that is in complete contrast with the isolation, loneliness, fear and suffering discussed in themes 1 and 2.

## 4. Discussion

The aim of this study was to describe the lived experiences and meaning-making of Russian-, Somali- and Arabic-speaking migrants in relation to COVID-19 and their responses to this threat in Finland during the first wave of pandemic. 

We identified three themes that shed light on respondents’ fears and negative experiences, and three themes that showed the different ways in which they coped with the situation. The seventh theme depicted respondents’ perceptions of the crisis as a globally shared burden, and how these perceptions capitalised on mutual dependency and equality. Our results support previous commentaries and studies on the migrant populations’ experiences of the COVID-19 pandemic, but they also add important insights that have previously been missed, as described below. 

In line with the large literature on the mental health effects of the COVID-19 pandemic [29], many of our respondents were anxious and scared. However, causes of this distress varied among individuals and groups. Traumatic experiences (e.g., expose to war and/or refugee experiences) might explain why health-related anxiety was especially pronounced among our Arabic-speaking respondents [3]. Most of them were immigrants from Iraq and a few from Syria (one from Palestine). Even though we did not ask how long the respondents had lived in Finland, the number of Iraqis in particular grew rapidly after 2015, and therefore it is likely that our sample included persons whose exposure to violent events was quite recent. The majority of the Somali population has settled in Finland already earlier. Media attention to the Arabic-speaking group due to the so-called refugee crisis may also have made them more sensitive to the media, and this may have influenced their level of stress; mainly Arabic-speaking migrants blamed the media for exaggerating the crisis. The media’s influence on coronavirus-related distress is already documented elsewhere [30].

Russian speakers were especially distressed about the economic consequences and uncertainty of the situation. In Finland, Russian speakers have a better socio-economic standing compared with Somali and Arabic speakers, many of whom have refugee backgrounds and face more discrimination in the job market [22]. Thus, the distress of our Russian-speaking respondents may reflect their ‘fear of falling’: the relatively wealthy are more concerned about economic instability than are those with lower incomes [31]. In addition, these respondents were younger than the other two groups, and so were likely more concerned about COVID-19′s social and societal effects than its health effects (we did not directly ask about respondents’ job market positions or educational backgrounds, because we wanted to avoid producing unnecessary stress during the interview. We knew that many respondents would be unemployed or living on other welfare subsidies, and this might be difficult to talk about by phone. During the interview, many respondents felt that even the question about their age was intrusive). Finally, they were the least confident in the Finnish authorities’ ability to manage the crisis, and the most critical of the Finnish healthcare system. Russian migrants’ low institutional trust and their suspicion of the Finnish healthcare system have also been demonstrated elsewhere (e.g., Pitkänen et al. [32]). Lack of trust in the authorities can make difficult to adapt to large-scale crises [33].

Our study supported the notion that there are many social factors that contribute to migrants’ vulnerability during the pandemic [3]. At the beginning of the crisis, our Arabic- and Somali-speaking respondents in particular did not know where to seek help, and information from the authorities did not reach them. Because they may have limited skills in the language of their host community and are unfamiliar with its official communication channels, migrants in general may be more dependent on social media that they are familiar with than host population. For the same reason they may more easily rely on informal oral communication when seeking information about their country of settlement. This can make some of them more susceptible to unreliable information. This might partly explain our findings among Arabic-speaking migrants.

Arabic- and Somali-speaking migrants were also seemingly worried about their transnational families, particularly in their countries of origin, which have suffered wars and long-term instability that have badly affected their healthcare systems. Our study also demonstrated that among the Somali speakers, migrants were worried about their community’s higher infection rates in Finland. Instead, explicit experiences of discrimination were little reported in our data. A few respondents regretted that information had entered the public domain about Somali-speaking migrants’ higher infection rates. In addition, one respondent was worried about suffering discrimination due to poor language skills when seeking help. It is important to note that we had not directly asked about this. 

As regards coping, our study reveals the role of sociocultural resources in coping with distress. This was especially evident among Somali-speaking respondents. Religion and community support seemed to provide them with special means to face adversity. However, during a pandemic, close social relationships can be a double-edged sword: they may partly explain the relatively high infection rates in the Somali community [34]. In addition, Somali speakers were the least critical of the Finnish authorities’ ability to manage the crisis, and many reported that the authorities tried to do their best and helped them. 

Most importantly, however, our study shows how migrants’ worries also turned into collective action aimed to support and protect their communities. This was particularly evident in the Somali sample. The reason why we did not observe similar moves towards collective action among Arabic and Russian speakers may be partly due to their lower infection rates and COVID-related stigmatisation, and also to the fact that the Arabic- and Russian-speaking communities are much more heterogeneous than Somali group in Finland.

We also found that the migrants’ experiences of collective action were not exclusively focused on supporting members of their own ethnic communities. As respondents from all migrant groups reported, cooperation with the Finnish authorities was the crucial part of the collective effort. In contrast with literature that has found increased social division and discrimination against ethnic minorities during the COVID-19 pandemic [9], our research demonstrates that COVID-19 can also provide opportunities to deepen cooperation and trust within migrant groups, and between those groups and the host society. Important factors seem to be perceived willingness to provide help and the understanding of mutual dependency [35]. However, these factors are insufficient if society does not have the material and institutional resources to handle the situation.

Our study has many practical implications for the management of the COVID-19 crisis in general and among migrant populations in particular. First, it confirms the importance of sharing reliable information and providing help in migrants’ native languages. Second, it demonstrates that migrant groups differ in their experiences of COVID-19 due to their different histories and societal positions. Thus, when measures are being planned to facilitate their situation, migrants’ socio-economic and culture-specific vulnerabilities and coping resources need to be taken into account. Third, it shows that migrants’ collective action and their cooperation with the authorities are important factors for alleviating distress and building cohesion in the fight against COVID-19 disease.

Although our study has many advantages (e.g., recruiting hard-to-reach respondents, studying their first-hand experiences, and comparing three different language groups), it is not without limitations. Since the data were collected via snowballing, it is possible that our sample was biased and self-selective in terms of demographics (e.g., education). It also needs to be noted that there were group differences in our data: our Russian-speaking respondents were younger than the other migrants, and the Russian and Somali speakers were mainly women. In addition, our Arabic- and Russian-speaking respondents came from many countries and/or ethnic groups and, therefore, these respondents had more heterogenous background than our Somali-speaking respondents did. Although these differences and our recruiting method might have affected our results, our findings seem to correspond in many ways with those made by many other researchers. However, it is important to acknowledge that no generalisations can be made based on the results of this study.

## 5. Conclusions

Our analysis shows that migrants constitute both a vulnerable and a resilient population in the COVID-19 pandemic in many ways. Migrant, and especially refugee background exposes people to stress and vulnerability. Thus, when the host society is under threat, these people can be in a particularly difficult situation. However, our analysis revealed that although our respondents reported fears and troubles, they also had many means to handle the situation both as individuals and groups. It demonstrated in particular that COVID-19 called for a deepening of cooperation within migrant groups, and between those groups, local authorities and professionals. Thus, our study suggests that cooperation, trust-building and effective information-sharing may foster functional adaptations to the COVID-19 pandemic and other future crises as well.

## Figures and Tables

**Table 1:** Gender, age, Finnish- and English-language skills, and whether respondents lived alone, by migrant group (*N* = 209).

Group	Arabic *N* = 65 ^a^		Russian *N* = 68		Somali *N* = 76		*χ*^2^ Test/*F*-Test	*p*-Value
	*N*	%	*N*	%	*N*	%		
Gender (female %)	33	51	57	84	59	78	20.01	*p* < 0.001
Age ^d^								
Under 40	5	8	7	10	1	1	29.76 ^b^	*p* < 0.001
40–49	16	25	28	41	4	5		
50–59	24	38	31	46	32	42		
60–69	11	16	2	3	30	40		
69+	7	11			9	12		
How well do you understand Finnish? ^c^								
Not at all	9	14	2	3	6	8	16.44 ^b^	*p* < 0.001
Badly	15	23	3	4	23	31		
Moderately	27	42	27	40	33	44		
Well	14	22	36	53	13	17		
How well do you understand English? ^d^								
Not at all	15	23	10	15	35	47	8.71 ^b^	*p* < 0.001
Badly	20	31	14	21	18	24		
Moderately	19	30	25	37	7	9		
Well	10	16	19	28	15	20		
Living alone (yes %)	20	31	8	12	7	9	13.47	*p* = 0.001

^a^ Includes one Iraq-born respondent who was interviewed in Finnish. ^b^ F-test. ^c^ One missing values. ^d^ Two missing values.

**Table 2:** Frequencies of subthemes and key themes per respondents.

Subtheme	Arabic f (%)	Russian f (%)	Somali f (%)	Key theme	Arabic f (%)	Russian f (%)	Somali f (%)
1. Coronavirus disease 2019 (COVID-19) as an unknown and dangerous health threat	29 (45)	17 (25)	28 (37)	1. COVID-19 infection as a health threat	41 (63)	22 (32)	32 (42)
2. Being worried about one’s own health	26 (40)	9 (13)	6 (8)				
1. COVID-19 as creating an exceptional and total crisis that causes fear and anxiety	8 (12)	24 (35)	6 (8)	2. Being vulnerable in Finland	38 (58)	52 (76)	40 (53)
2. Being distressed and worried about social, societal and economic consequences of the crisis	8 (12)	23 (34)	9 (12)				
3. Distrust in Finnish society’s ability to handle the crisis	15 (23)	23 (34)	9 (12)				
4. Lack of the resources, abilities and support needed to cope with the crisis	24 (37)	5 (7)	23 (30)				
1. Being worried about friends and relatives in Finland and elsewhere	30 (46)	30 (44)	10 (13)	3. Communal and multilocal worries	49 (75)	32 (47)	72 (95)
2. Being worried about one’s ethnic/language community in Finland (and Sweden)	0 (0)	4 (6)	18 (24)				
3. Feeling personal concern about the situation in multiple countries at the same time	33 (51)	2 (3)	69 (91)				
1. Preserving peace of mind	24 (37)	20 (29)	60 (79)	4. Living with the threat of the virus in the everyday	65 (100)	68 (100)	76 (100)
2. Taking care of oneself and doing one’s best	64 (98)	67 (99)	71 (93)				
3. Avoidance	15 (23)	6 (9)	0 (0)				
4. Downplaying the COVID-19 threat and blaming the media	30 (46)	5 (7)	2 (3)				
1. Being in need and asking for help from the authorities	34 (52)	52 (76)	53 (70)	5. Building trust and cooperation with Finnish society	51 (78)	59 (87)	71 (93)
2. Good will of the authorities, and Finland’s good resources	34 (52)	38 (56)	57 (75)				
3. Cooperation between citizens/residents and authorities	20 (31)	25 (37)	36 (47)				
1. Family and friends as supportive	56 (86)	62 (91)	66 (87)	6. Supporting each other and taking collective action	58 (89)	62 (91)	71 (93)
2. Acting as a community	16 (25)	1 (1)	35 (46)				
1. Globally shared worry and equality among people	10 (15)	17 (25)	31 (41)	7. Globally shared worry and cross-border solidarity	14 (22)	27 (40)	40 (53)
2. Solidarity	5 (8)	13 (19)	12 (16)				

## Data Availability

The data presented in this study are available on request from the corresponding author. The data are not publicly available because of securing the anonymity of the respondents.

## Preprint

The manuscript is preprinted (see https://psyarxiv.com/q2v7r/, doi: https://psyarxiv.com/q2v7r/) (accessed on 23 November 2020).

## Appendix A

**Table A1:** List of key codes.

Key Code	Subtheme	Key Theme
Spreads easily	COVID-19 as an unknown and dangerous health threat	*COVID-19 infection as a health threat*
No medicine nor vaccine		
Similar symptoms to flu		
Dangerous virus		
Unknown and threatening virus		
General worry about one’s health	Being worried about one’s own health	
Being afraid of becoming infected, and fear about symptoms		
Belonging to a risk group		
Distressing uncertainty	COVID-19 as creating an exceptional and total crisis that causes fear and anxiety	*Being vulnerable in Finland*
Finding the whole situation very stressful		
Unexpected situation		
Being worried about societal stability	Being distressed and worried about social, societal and economic consequences of the crisis	
Being worried about the economic and employment situation		
Being distressed about being socially isolated		
Distrust in Finnish authorities’ and residents’ ability to handle the crisis	Distrust in Finnish society’s ability to handle the crisis	
Distrust in the Finnish health service		
Lacking language and other skills	Lack of the resources, abilities and support needed to cope with the crisis	
Lacking support from social networks and Finnish society		
Lacking knowledge about (Finnish authorities’) information and where to seek help		
Worry about friends and family	Being worried about friends and relatives in Finland and elsewhere	*Communal and multilocal worries*
Worry about friends and family in other countries		
Being worried about one’s community	Being worried about one’s ethnic/language community in Finland (and Sweden)	
Asking for more information for others		
Being worried about the situation in one’s country of origin	Feeling personal concern about the situation in multiple countries at the same time	
Being worried about Finland and one’s country of origin		
Avoiding panic	Preserving peace of mind	*Living with the threat of the virus in the everyday*
Accepting one’s destiny		
Trusting in God and being a good Muslim		
Taking responsibility for oneself	Taking care of oneself and doing one’s best	
One needs to do one’s best		
I take care of myself		
Taking care of my hygiene and/or avoiding contact		
Taking care of my nutrition only		
Avoiding thinking	Avoidance	
Avoiding the news		
(Social) media and other people exaggerate the threat and cause unnecessary stress	Downplaying the COVID-19 threat and blaming the media	
There is nothing to be afraid of, I live a normal life		
It is made by humans, so no worry		
It is like any other virus		
Asking for more information for oneself	Being in need and asking for help from the authorities	*Building trust and cooperation with Finnish society*
Need medical help and psychological support		
Need economic help		
Need help with protective equipment		
Need help with food and other practical things		
Seeking help from the authorities		
Authorities are doing their best	Good will of the authorities, and Finland’s good resources	
Authorities are providing help		
Finland is doing better than other countries		
Quality of healthcare is high		
Authorities can handle the epidemic		
Good resources in a small country		
Trust that residents are following the instructions	Cooperation between citizens/residents and authorities	
It is important that residents cooperate and follow the recommendations		
I follow the recommendations		
Family members and friends in Finland and abroad share information with me	Family and friends as supportive	*Supporting each other and taking collective action*
I receive help and support from my family and friends		
I help and provide support to my family and friends		
My community is sharing information and support	Acting as a community	
I share information and support with my own community		
Responsibility for one’s community		
This is a shared experience Global worry	Globally shared worry and equality among people	*Globally shared worry and cross-border solidarity*
We need help each other Worry about all (vulnerable) people	Solidarity	
